# Supplementary material for: Use of an extended KDIGO definition to diagnose acute kidney injury in patients with COVID-19: A multinational study using the ISARIC–WHO clinical characterisation protocol
Source: PLoS Med. 2022 Apr 20;19(4):e1003969. doi: 10.1371/journal.pmed.1003969 (PMC9067700; doi:10.1371/journal.pmed.1003969)
Supplement: S1 Statement — (DOCX) [file pmed.1003969.s001.docx]

**S1 Statement.** Study ethics approval

Ethics Committee approval for this work was given by the World Health Organisation Ethics Review Committee (RPC571 and RPC572 on 25 April 2013). Institutional approval was additionally obtained by participating sites including the South Central Oxford C Research Ethics Committee in England (Ref 13/SC/0149) and the Scotland A Research Ethics Committee (Ref 20/SS/0028) for the United Kingdom and the Human Research Ethics Committee (Medical) at the University of the Witwatersrand in South Africa as part of a national surveillance programme (M160667) collectively representing the majority of the data. Other institutional and national approvals are in place as per local requirements.
