# Supplementary material for: Use of an extended KDIGO definition to diagnose acute kidney injury in patients with COVID-19: A multinational study using the ISARIC–WHO clinical characterisation protocol
Source: PLoS Med. 2022 Apr 20;19(4):e1003969. doi: 10.1371/journal.pmed.1003969 (PMC9067700; doi:10.1371/journal.pmed.1003969)
Supplement: S1 Table — COVID-19, Coronavirus Disease 2019. (DOCX) [file pmed.1003969.s002.docx]

**S1 Table.** Definitions used for clinical COVID-19

| Source of definition | Definition for clinical COVID-19 |
| --- | --- |
| World Health Organization (WHO) | A combination of acute fever and cough,  Or  A combination of three or more of: fever, cough, general weakness and fatigue, headache, myalgia, sore throat, coryza, dyspnoea, anorexia, nausea and vomiting, diarrhoea, altered mental status |
| Centers for Disease Control (CDC), United States | At least two of: fever, chills*, rigors *, myalgia, headache, sore throat, new olfactory and taste disorder,  Or  At least one of: cough, shortness of breath, difficulty breathing* |
| Public Health England | New cough, or temperature 37.8°C, or a loss or change in sense of smell or taste |
| European Centre for Disease Prevention and Control | At least one of: cough, fever, shortness of breath, sudden onset anosmia, ageusia or dysgeusia |

* Symptom information not collected in the Case Report Form (CRF)
