# Supplementary material for: Use of an extended KDIGO definition to diagnose acute kidney injury in patients with COVID-19: A multinational study using the ISARIC–WHO clinical characterisation protocol
Source: PLoS Med. 2022 Apr 20;19(4):e1003969. doi: 10.1371/journal.pmed.1003969 (PMC9067700; doi:10.1371/journal.pmed.1003969)
Supplement: S2 Table — ISARIC, International Severe Acute Respiratory and Emerging Infection Consortium; CRF, case report form. (DOCX) [file pmed.1003969.s003.docx]

**S2 Table.** Definition of comorbidities, complications and outcomes from the ISARIC case report forms (CRF)

| Comorbidity | Definition |
| --- | --- |
| Chronic cardiac disease | Any of coronary artery disease, heart failure, congenital heart disease, cardiomyopathy, rheumatic heart disease. |
| Hypertension | Elevated arterial blood pressure diagnosed clinically, >140mmHg systolic or >90mmHg diastolic. |
| Chronic pulmonary disease (not asthma) | Any of chronic obstructive pulmonary disease (chronic bronchitis, chronic obstructive pulmonary disease (COPD), emphysema), cystic fibrosis, bronchiectasis, interstitial lung disease, pre-existing requirement for long term oxygen therapy. |
| Chronic kidney disease | Clinician-diagnosed chronic kidney disease, chronic estimated glomerular filtration rate < 60 mL/min/1.73m2, history of kidney transplantation |
| Obesity | Patients for whom an attending clinician has assessed them to be obese - ideally but not necessarily with an objective measurement of obesity, such as calculation of the body mass index (BMI of 30 or more) or measurement of abdominal girth. |
| Liver disease (mild, moderate & severe) | Cirrhosis with or without portal hypertension or chronic hepatitis, with or without bleeding or a history of variceal bleeding |
| Type 2 Diabetes | Clinician diagnosed requiring oral or subcutaneous treatment |
| Dementia | Clinical diagnosis of dementia |
| Malnutrition | Any clinically identified deficiency in intake, either of total energy or of specific nutrients that led to a dietetic intervention or referral prior to the onset of COVID-19 symptoms. Does not include people who needed supplementary nutrition solely due to reduced intake during their current illness episode. |
| Complication | |
| Bacterial pneumonia | Clinically or radiologically diagnosed bacterial pneumonia (including community, hospital and ventilator acquired) managed with antimicrobials. Bacteriological confirmation not required. |
| Cardiac arrest | Sudden cessation of cardiac activity with no normal breathing and no signs of circulation. |
| Coagulation disorder | Abnormal coagulation identified by abnormal prothrombin time or activated partial thromboplastin time. Disseminated intravascular coagulation (DIC; consumption coagulopathy; defibrination syndrome) is defined by thrombocytopenia, prolonged prothrombin time, low fibrinogen, elevated D-dimer and thrombotic microangiopathy. |
| Rhabdomyolysis | Rhabdomyolysis is a syndrome characterised by muscle necrosis and the release of myoglobin into the blood. Muscle biopsy, electromyography, radiological imaging and the presence of myoglobinuria are not required for the diagnosis. |
| Acute kidney injury | Acute renal injury is defined as any of:   - Increase in serum creatinine by ≥0.3 mg/dL (≥26.5 μmol/L) within 48 hours - Increase in serum creatinine to ≥1.5 times baseline, which is known or presumed to have occurred within the prior 7 days - Urine volume <0.5 mL/kg/hour for 6 hours |
| Outcomes | |
| ICU admission | Admission to an intensive care unit (ICU) or high dependency care unit (HDU) |
| Invasive mechanical ventilation | Any mechanical ventilation delivered following intubation or via a tracheostomy. Does not include patients who are breathing independently via a tracheostomy. |
| Length of stay | Number of days in hospital up until discharge or study censoring date |
| Still in hospital | Patient is still in hospital at the time of the study censoring date |
| Transferred | Patient has been transferred to another facility that provides medical care. This could be a specialist centre for more intensive treatment or a step-down for rehabilitation. It does not include facilities that solely provide social care (these patients should be listed as discharged alive). |
| Discharged alive | Patient has been discharged to their usual place of residence before their illness, to the home of a relative or friend, or to a social care facility, because their illness is no longer severe enough to warrant treatment in a medical facility. |
| Death | Patient died in the hospital |
