# Supplementary material for: Use of an extended KDIGO definition to diagnose acute kidney injury in patients with COVID-19: A multinational study using the ISARIC–WHO clinical characterisation protocol
Source: PLoS Med. 2022 Apr 20;19(4):e1003969. doi: 10.1371/journal.pmed.1003969 (PMC9067700; doi:10.1371/journal.pmed.1003969)
Supplement: S3 Table — AKI, acute kidney injury; eKDIGO, extended KDIGO. (DOCX) [file pmed.1003969.s004.docx]

**S3 Table.** Distribution of missingness information between eKDIGO and No AKI patients.

|  |  | **deKDIGO Missing (%)** | **KDIGO Missing (%)** | **eKDIGO Missing (%)** | **No AKI Missing (%)** |
| --- | --- | --- | --- | --- | --- |
| **Total Count** | | | | | |
|  |  | 11188 | 12704 | 23892 | 51772 |
| **Demographics** | | | | | |
|  | Age | 26 (0.23) | 63 (0.5) | 89 (0.37) | 35 (0.07) |
|  | Female | 53 (0.47) | 91 (0.72) | 144 (0.6) | 125 (0.24) |
| **Country Income Level, n (%)** | | | | | |
|  | High Income | 26 (0.23) | 63 (0.5) | 89 (0.37) | 34 (0.07) |
|  | Upper Middle Income | 26 (0.23) | 63 (0.5) | 89 (0.37) | 34 (0.07) |
|  | Low & low middle income | 26 (0.23) | 63 (0.5) | 89 (0.37) | 34 (0.07) |
| **AKI Grades & RRT, n (%)** |  | | | | |
|  | AKI Stage 1 | 0 (0.0) | 0 (0.0) | 0 (0.0) | 0 (0.0) |
|  | AKI Stage 2 | 0 (0.0) | 0 (0.0) | 0 (0.0) | 0 (0.0) |
|  | AKI Stage 3* | 0 (0.0) | 0 (0.0) | 0 (0.0) | 0 (0.0) |
|  | RRT | 673 (6.02) | 752 (5.92) | 1425 (5.96) | 5385 (10.4) |
| **Comorbidities**, n (%)** | | | | | |
|  | Chronic Kidney Disease | 744 (6.65) | 998 (7.86) | 1742 (7.29) | 3819 (7.38) |
|  | Chronic Cardiac Disease | 184 (1.64) | 256 (2.02) | 440 (1.84) | 1235 (2.39) |
|  | Chronic Pulmonary Disease | 716 (6.4) | 1010 (7.95) | 1726 (7.22) | 3659 (7.07) |
|  | Hypertension | 3127 (27.95) | 3868 (30.45) | 6995 (29.28) | 13858 (26.77) |
|  | Dementia | 1111 (9.93) | 1737 (13.67) | 2848 (11.92) | 6066 (11.72) |
|  | Diabetes -type 2 | 611 (5.46) | 895 (7.05) | 1506 (6.3) | 3207 (6.19) |
|  | Liver disease | 351 (3.14) | 765 (6.02) | 1116 (4.67) | 1963 (3.79) |
|  | Malnutrition | 1337 (11.95) | 1456 (11.46) | 2793 (11.69) | 6431 (12.42) |
|  | Obesity | 1821 (16.28) | 2218 (17.46) | 4039 (16.91) | 9509 (18.37) |
| **Medications on Admission, n (%)** | | | | | |
|  | NSAIDS | 3549 (31.72) | 4557 (35.87) | 8106 (33.93) | 18172 (35.1) |
|  | ACEi | 3381 (30.22) | 4349 (34.23) | 7730 (32.35) | 17487 (33.78) |
|  | ARBs | 3393 (30.33) | 4342 (34.18) | 7735 (32.37) | 17575 (33.95) |
| **Signs & Symptoms on Admission, n (%)** | | | | | |
|  | Altered Consciousness / Confusion | 2241 (20.03) | 2595 (20.43) | 4836 (20.24) | 9589 (18.52) |
|  | Diarrhea | 2382 (21.29) | 2524 (19.87) | 4906 (20.53) | 9263 (17.89) |
|  | Fever | 1610 (14.39) | 1606 (12.64) | 3216 (13.46) | 5657 (10.93) |
|  | Vomiting /nausea | 2364 (21.13) | 2560 (20.15) | 4924 (20.61) | 9173 (17.72) |
|  | Muscle aches/joint pain | 3228 (28.85) | 3335 (26.25) | 6563 (27.47) | 12651 (24.44) |
|  | Headache | 3315 (29.63) | 3375 (26.57) | 6690 (28.0) | 12846 (24.81) |
|  | Sore Throat | 3443 (30.77) | 3583 (28.2) | 7026 (29.41) | 13667 (26.4) |
|  | Cough | 1709 (15.28) | 1763 (13.88) | 3472 (14.53) | 5973 (11.54) |
|  | Cough, with sputum | 2848 (25.46) | 2976 (23.43) | 5824 (24.38) | 10841 (20.94) |
|  | Cough, with bloody sputum | 3019 (26.98) | 3320 (26.13) | 6339 (26.53) | 12145 (23.46) |
|  | Shortness of breath | 1620 (14.48) | 1580 (12.44) | 3200 (13.39) | 5940 (11.47) |
|  | Runny nose | 3517 (31.44) | 3618 (28.48) | 7135 (29.86) | 14143 (27.32) |
| **Observations on Admission, n (%)** | | | | | |
|  | Temperature | 244 (2.18) | 527 (4.15) | 771 (3.23) | 1362 (2.63) |
|  | Systolic BP | 357 (3.19) | 540 (4.25) | 897 (3.75) | 3437 (6.64) |
|  | Diastolic | 491 (4.39) | 665 (5.23) | 1156 (4.84) | 3932 (7.59) |
|  | Heart rate | 416 (3.72) | 762 (6.0) | 1178 (4.93) | 4259 (8.23) |
|  | Respiratory rate | 1316 (11.76) | 1561 (12.29) | 2877 (12.04) | 7120 (13.75) |
|  | Oxygen saturation | 475 (4.25) | 822 (6.47) | 1297 (5.43) | 3906 (7.54) |
|  | WBC | 1055 (9.43) | 1903 (14.98) | 2958 (12.38) | 8031 (15.51) |
|  | BUN | 1913 (17.1) | 2331 (18.35) | 4244 (17.76) | 12508 (24.16) |
|  | Potassium | 1000 (8.94) | 1682 (13.24) | 2682 (11.23) | 8963 (17.31) |
|  | CRP | 1720 (15.37) | 3294 (25.93) | 5014 (20.99) | 11861 (22.91) |
|  | Admission sCr | 637 (5.69) | 1226 (9.65) | 1863 (7.8) | 6720 (12.98) |
|  | Admission eGFR | 660 (5.9) | 1252 (9.86) | 1912 (8.0) | 6796 (13.13) |
| **Admission Treatment, n (%)** | | | | | |
|  | Antiviral and COVID-19 targeting agents | 1743 (15.58) | 1945 (15.31) | 3688 (15.44) | 8477 (16.37) |
|  | Antibiotic agents | 542 (4.84) | 851 (6.7) | 1393 (5.83) | 4788 (9.25) |
|  | Antifungal agents | 872 (7.79) | 1351 (10.63) | 2223 (9.3) | 5859 (11.32) |
|  | Corticosteroids | 696 (6.22) | 931 (7.33) | 1627 (6.81) | 5045 (9.74) |
| **Complications**, n (%)** | | | | | |
|  | Bacterial pneumonia | 1474 (13.17) | 1934 (15.22) | 3408 (14.26) | 8885 (17.16) |
|  | Cardiac arrest | 860 (7.69) | 1121 (8.82) | 1981 (8.29) | 5515 (10.65) |
|  | Coagulation disorder | 1346 (12.03) | 1996 (15.71) | 3342 (13.99) | 8190 (15.82) |
|  | Rhabdomyolysis | 1311 (11.72) | 1973 (15.53) | 3284 (13.75) | 8107 (15.66) |
| **Outcomes, n (%)** | | | | | |
|  | ICU admission | 262 (2.34) | 252 (1.98) | 514 (2.15) | 1291 (2.49) |
|  | Invasive mechanical ventilation | 375 (3.35) | 537 (4.23) | 912 (3.82) | 4141 (8.0) |
|  | Length of Stay (median, IQR) | 415 (3.71) | 653 (5.14) | 1068 (4.47) | 1752 (3.38) |
|  | Still in hospital | 271 (2.42) | 395 (3.11) | 666 (2.79) | 906 (1.75) |
|  | Transferred | 271 (2.42) | 395 (3.11) | 666 (2.79) | 906 (1.75) |
|  | Discharged | 271 (2.42) | 395 (3.11) | 666 (2.79) | 906 (1.75) |
|  | Death | 271 (2.42) | 395 (3.11) | 666 (2.79) | 906 (1.75) |

*Stage 3 includes patients requiring RRT for KDIGO and eKDIGO

** Definitions of comorbidities, complications and outcomes from the CRFs are presented in S2 Table

RRT = Renal replacement therapy; ACEi = Angiotensin converting enzyme inhibitors; ARBs = Angiotensin II receptor blockers; NSAIDs = non-steroidal anti-inflammatories; BUN = Blood urea nitrogen; CRP = C-reactive protein; WBC = White blood cell; sCr = serum creatinine; eGFR = estimated glomerular filtration rate (estimated using the CKD-EPI equation)
