# Supplementary material for: Use of an extended KDIGO definition to diagnose acute kidney injury in patients with COVID-19: A multinational study using the ISARIC–WHO clinical characterisation protocol
Source: PLoS Med. 2022 Apr 20;19(4):e1003969. doi: 10.1371/journal.pmed.1003969 (PMC9067700; doi:10.1371/journal.pmed.1003969)
Supplement: S4 Table — AKI, acute kidney injury; eKDIGO, extended KDIGO; MICE, Multiple Imputation by Chained Equations. (DOCX) [file pmed.1003969.s005.docx]

**S4 Table.** Logistic regression fitted to assess the association between eKDIGO AKI with in-hospital mortality without MICE imputation for variable missingness

|  |  | **95% Confidence Interval** | |  |
| --- | --- | --- | --- | --- |
| **Variable** | **Odds Ratio** | **Lower** | **Upper** | **P value** |
| **(Intercept)** | 0.048 | 0.045 | 0.051 | < 0.001 |
| **AKI eKDIGO** | 1.785 | 1.714 | 1.859 | < 0.001 |
| **Age 18 – 65 (ref)** | 1.0 | - | - | - |
| **Age 65 - 85** | 3.926 | 3.739 | 4.123 | < 0.001 |
| **Age 85+** | 7.652 | 7.174 | 8.164 | < 0.001 |
| **Female TRUE** | 0.784 | 0.754 | 0.816 | < 0.001 |
| **Female UNKNOWN** | 0.643 | 0.396 | 1.011 | 0.064 |
| **Chronic Kidney Disease TRUE** | 1.282 | 1.214 | 1.353 | < 0.001 |
| **Chronic Kidney Disease UNKNOWN** | 0.951 | 0.821 | 1.100 | 0.497 |
| **Chronic Cardiac Disease Unknown** | 1.330 | 1.273 | 1.390 | < 0.001 |
| **Chronic Cardiac Disease TRUE** | 0.804 | 0.673 | 0.959 | 0.016 |
| **Chronic Pulmonary Disease UNKNOWN** | 1.495 | 1.418 | 1.576 | < 0.001 |
| **Chronic Pulmonary Disease TRUE** | 1.000 | 0.864 | 1.157 | 0.999 |
| **Hypertension TRUE** | 0.966 | 0.919 | 1.015 | 0.168 |
| **Hypertension UNKNOWN** | 1.149 | 1.046 | 1.263 | 0.004 |
| **Diabetes Type 2 Unknown** | 1.209 | 1.156 | 1.264 | < 0.001 |
| **Diabetes Type 2 TRUE** | 0.979 | 0.853 | 1.122 | 0.757 |
| **Obesity TRUE** | 0.921 | 0.867 | 0.979 | 0.008 |
| **Obesity UNKNOWN** | 1.073 | 1.005 | 1.145 | 0.034 |
| **ACEi & ARBs TRUE** | 0.868 | 0.820 | 0.918 | < 0.001 |
| **ACEi & ARBs UNKNOWN** | 0.936 | 0.869 | 1.009 | 0.084 |
| **Corticosteroids UNKNOWN** | 1.173 | 1.122 | 1.226 | < 0.001 |
| **Corticosteroids TRUE** | 1.309 | 1.157 | 1.481 | < 0.001 |
| **Antifungal Agents UNKNOWN** | 1.222 | 1.138 | 1.312 | < 0.001 |
| **Antifungal Agents TRUE** | 0.913 | 0.813 | 1.026 | 0.126 |
| **ICU Admission TRUE** | 1.795 | 1.682 | 1.914 | < 0.001 |
| **ICU Admission UNKNOWN** | 1.531 | 1.323 | 1.770 | < 0.001 |
| **Mechanical Ventilation TRUE** | 1.968 | 1.836 | 2.109 | < 0.001 |
| **Mechanical Ventilation UNKNOWN** | 0.393 | 0.348 | 0.445 | < 0.001 |
| **Bacterial Pneumonia TRUE** | 1.219 | 1.154 | 1.287 | < 0.001 |
| **Bacterial Pneumonia UNKNOWN** | 1.226 | 1.115 | 1.347 | < 0.001 |
| **Cardiac Arrest TRUE** | 20.390 | 17.880 | 23.339 | < 0.001 |
| **Cardiac Arrest UNKNOWN** | 3.580 | 3.166 | 4.049 | < 0.001 |
| **Coagulation UNKNOWN** | 1.344 | 1.223 | 1.477 | < 0.001 |
| **Coagulation TRUE** | 0.747 | 0.621 | 0.896 | 0.002 |
| **Rhabdomyolysis TRUE** | 1.138 | 0.910 | 1.418 | 0.255 |
| **Rhabdomyolysis UNKNOWN** | 0.604 | 0.498 | 0.733 | < 0.001 |

ACEi: Angiotensin converting enzyme inhibitors; ARBs: Angiotensin receptor blockers
