# Supplementary material for: Use of an extended KDIGO definition to diagnose acute kidney injury in patients with COVID-19: A multinational study using the ISARIC–WHO clinical characterisation protocol
Source: PLoS Med. 2022 Apr 20;19(4):e1003969. doi: 10.1371/journal.pmed.1003969 (PMC9067700; doi:10.1371/journal.pmed.1003969)
Supplement: S5 Table — STROBE, STrengthening the Reporting of OBservational studies in Epidemiology. (DOCX) [file pmed.1003969.s006.docx]

**S5 Table.** STROBE (Strengthening the reporting of observational studies in epidemiology) checklist

|  | Item No | Recommendation | Section & Paragraph |
| --- | --- | --- | --- |
| **Title and abstract** | 1 | (*a*) Indicate the study’s design with a commonly used term in the title or the abstract | Title & abstract as per PloS guidelines |
|  |  | (*b*) Provide in the abstract an informative and balanced summary of what was done and what was found |  |
| Introduction | | | |
| Background/rationale | 2 | Explain the scientific background and rationale for the investigation being reported | Intro, para 1-3 |
| Objectives | 3 | State specific objectives, including any prespecified hypotheses | Intro para 4 |
| Methods | | | |
| Study design | 4 | Present key elements of study design early in the paper | Methods Study Design 1-2 |
| Setting | 5 | Describe the setting, locations, and relevant dates, including periods of recruitment, exposure, follow-up, and data collection | **Study Population**. Inclusion & Exclusion Criteria para 1.  **Data Collection & time to peak AKI** para 1&3 |
| Participants | 6 | (*a*) Give the eligibility criteria, and the sources and methods of selection of participants. Describe methods of follow-up | **a) Study Population**. Inclusion & Exclusion Criteria para 1. Fig 1 |
|  |  | (*b*) For matched studies, give matching criteria and number of exposed and unexposed |  |
| Variables | 7 | Clearly define all outcomes, exposures, predictors, potential confounders, and effect modifiers. Give diagnostic criteria, if applicable | Methods. **AKI Definitions & Diagnosis** para 1 + Table 1 |
| Data sources/ measurement | 8* | For each variable of interest, give sources of data and details of methods of assessment (measurement). Describe comparability of assessment methods if there is more than one group | **Data collection and time to peak AKI** para 1 – 3  **Statistical Analysis** Para 1-2 |
| Bias | 9 | Describe any efforts to address potential sources of bias | - |
| Study size | 10 | Explain how the study size was arrived at | **Study Population**. Inclusion & Exclusion Criteria para 1. Fig 1 |
| Quantitative variables | 11 | Explain how quantitative variables were handled in the analyses. If applicable, describe which groupings were chosen and why | **Statistical Analysis** para 1-2 |
| Statistical methods | 12 | (*a*) Describe all statistical methods, including those used to control for confounding | a) **Statistical Analysis** para 1-4, b) **Statistical Analysis** para 1 & **Data collection and Time to Peak AKI** para 2, c) **Statistical analysis** para 2, d) **Statistical analysis** para 2, e) **Statistical analysis** para 2 |
|  |  | (*b*) Describe any methods used to examine subgroups and interactions |  |
|  |  | (*c*) Explain how missing data were addressed |  |
|  |  | (*d*) If applicable, explain how loss to follow-up was addressed |  |
|  |  | (*e*) Describe any sensitivity analyses |  |
| Results | | |  |
| Participants | 13* | (a) Report numbers of individuals at each stage of study—eg numbers potentially eligible, examined for eligibility, confirmed eligible, included in the study, completing follow-up, and analysed | a) **Results** para 1 & Fig 1 + **Methods Study Population Inc & Exc criteria** para 1  b) as per point a)  c) Fig 1 |
|  |  | (b) Give reasons for non-participation at each stage |  |
|  |  | (c) Consider use of a flow diagram |  |
| Descriptive data | 14* | (a) Give characteristics of study participants (eg demographic, clinical, social) and information on exposures and potential confounders | a) **Results, Demographic & Clinical characteristics** para 1 & 2 (Tables 2,3,4)  b) **Results** para 1, Tables 2, 3, 4 (missingness)  c) Results para 1 |
|  |  | (b) Indicate number of participants with missing data for each variable of interest |  |
|  |  | (c) Summarise follow-up time (eg, average and total amount) |  |
| Outcome data | 15* | Report numbers of outcome events or summary measures over time | **Results Clinical Outcomes** para 1, Tables 2, 3 and 4 |
