# Supplementary material for: Use of an extended KDIGO definition to diagnose acute kidney injury in patients with COVID-19: A multinational study using the ISARIC–WHO clinical characterisation protocol
Source: PLoS Med. 2022 Apr 20;19(4):e1003969. doi: 10.1371/journal.pmed.1003969 (PMC9067700; doi:10.1371/journal.pmed.1003969)
Supplement: S1 Fig — AKI, acute kidney injury; KDIGO, Kidney Disease Improving Global Outcomes. (DOCX) [file pmed.1003969.s007.docx]

**S1 Fig.** Breakdown of top contributing countries for patients diagnosed with AKI by KDIGO definition (A) and from deKDIGO group (B)

A*

B*

* Y-axis has logarithmic scaling
