# Supplementary material for: Use of an extended KDIGO definition to diagnose acute kidney injury in patients with COVID-19: A multinational study using the ISARIC–WHO clinical characterisation protocol
Source: PLoS Med. 2022 Apr 20;19(4):e1003969. doi: 10.1371/journal.pmed.1003969 (PMC9067700; doi:10.1371/journal.pmed.1003969)
Supplement: S2 Fig — AKI, acute kidney injury; eKDIGO, extended KDIGO; KDIGO, Kidney Disease Improving Global Outcomes; sCr, serum creatinine. (DOCX) [file pmed.1003969.s008.docx]

**S2 Fig.** Number of AKI cases by AKI definition (A = KDIGO and B = eKDIGO) as a proportion of total number of serum creatinines collected each day

A

B

* sCr = serum creatinine
