# Supplementary material for: Use of an extended KDIGO definition to diagnose acute kidney injury in patients with COVID-19: A multinational study using the ISARIC–WHO clinical characterisation protocol
Source: PLoS Med. 2022 Apr 20;19(4):e1003969. doi: 10.1371/journal.pmed.1003969 (PMC9067700; doi:10.1371/journal.pmed.1003969)
Supplement: S1 Acknowledgements — ISARIC, International Severe Acute Respiratory and Emerging Infection Consortium. (DOCX) [file pmed.1003969.s009.docx]

**S1 Acknowledgements**

The ISARIC Clinical Characterisation Group includes all data contributors as well as individuals involved in data curation, supervision, resources, project administration, methodology, formal analysis, funding acquisition, software, validation, visualization, and conceptualization: Ali Abbas, Sheryl Ann Abdukahil, Nurul Najmee Abdulkadir, Ryuzo Abe, Laurent Abel, Lara Absil, Subhash Acharya, Andrew Acker, Shingo Adachi, Diana Adrião, Saleh Al Ageel, Quratul Ain, Kate Ainscough, Ali Ait Hssain, Younes Ait Tamlihat, Takako Akimoto, Ernita Akmal, Eman Al Qasim, Razi Alalqam, Tanvir Alam, Tala Al-dabbous, Cynthia Alegre, Marta Alessi, Beatrice Alex, Kévin Alexandre, Abdulrahman Al-Fares, Huda Alfoudri, Naseem Ali Shah, Kazali Enagnon Alidjnou, Jeffrey Aliudin, Clotilde Allavena, Nathalie Allou, Aneela Altaf, João Alves, João Melo Alves, Rita Alves, Maria Amaral, Nur Amira, Heidi Ammerlaan, Beatriz Amorim Beltrão, Phoebe Ampaw, Roberto Andini, Claire Andrejak, Andrea Angheben, François Angoulvant, Séverine Ansart, Sivanesen Anthonidass, Massimo Antonelli, Carlos Alexandre Antunes de Brito, Ardiyan Apriyana, Carolline Araujo, Yaseen Arabi, Irene Aragao, Francisco Arancibia, Antonio Arcadipane, Patrick Archambault, Lukas Arenz, Jean-Benoît Arlet, Christel Arnold-Day, Lovkesh Arora, Rakesh Arora, Elise Artaud-Macari, Diptesh Aryal, Angel Asensio, Elizabeth Ashley, Muhammad Ashraf, Mohammad Asim, Jean Baptiste Assie, Amirul Asyraf, Anika Atique, AM Udara Lakshan Attanyake, Johann Auchabie, Hugues Aumaitre, Adrien Auvet, Laurène Azemar, Cecile Azoulay, Delphine Bachelet, Claudine Badr, Nadia Baig, J. Kenneth Baillie, Erica Bak, Agamemnon Bakakos, Nazreen Abu Bakar, Andriy Bal, Mohanaprasanth Balakrishnan, Firouzé Bani-Sadr, Renata Barbalho, Wendy S. Barclay, Saef Umar Barnett, Michaela Barnikel, Helena Barrasa, Audrey Barrelet, Cleide Barrigoto, Marie Bartoli, Cheryl Bartone, Joaquín Baruch, Romain Basmaci, Muhammad Fadhli Hassin Basri, Denise Battaglini, Jules Bauer, Diego Fernando Bautista Rincon, Abigail Beane, Alexandra Bedossa, Ker Hong Bee, Husna Begum, Sylvie Behilill, Albertus Beishuizen, Aleksandr Beljantsev, Anna Beltrame, Marine Beluze, Nicolas Benech, Lionel Eric Benjiman, Dehbia Benkerrou, Suzanne Bennett, Luís Bento, Jan-Erik Berdal, Delphine Bergeaud, José Luis Bernal Sobrino, Giulia Bertoli, Lorenzo Bertolino, Simon Bessis, Sybille Bevilcaqua, Karine Bezulier, Amar Bhatt, Krishna Bhavsar, Isabella Bianchi, Claudia Bianco, Farah Nadiah Bidin, Moirangthem Bikram Singh, Felwa Bin Humaid, Mohd Nazlin Bin Kamarudin, François Bissuel, Patrick Biston, Laurent Bitker, Pablo Blanco-Schweizer, Frank Bloos, Mathieu Blot, Lucille Blumberg, Filomena Boccia, Laetitia Bodenes, Alice Bogaarts, Debby Bogaert, Anne-Hélène Boivin, Pierre-Adrien Bolze, François Bompart, Diogo Borges, Raphaël Borie, Elisabeth Botelho-Nevers, Lila Bouadma, Olivier Bouchaud, Sabelline Bouchez, Dounia Bouhmani, Damien Bouhour, Kévin Bouiller, Laurence Bouillet, Camile Bouisse, Anne-Sophie Boureau, Maude Bouscambert, Aurore Bousquet, Jason Bouziotis, Bianca Boxma, Marielle Boyer-Besseyre, Maria Boylan, Fernando Augusto Bozza, Matthew Brack, Axelle Braconnier, Cynthia Braga, Filipa Brás Monteiro, Luca Brazzi, Dorothy Breen, Patrick Breen, Kathy Brickell, Tessa Broadley, Marjolein Brusse-Keizer, Nina Buchtele, Christian Buesaquillo, Polina Bugaeva, Marielle Buisson, Erlina Burhan, Aidan Burrell, Ingrid G. Bustos, Denis Butnaru, André Cabie, Susana Cabral, Eder Caceres, Cyril Cadoz, Jose Andres Calvache, João Cam, Valentine Campana, Paul Campbell, Mireia Cantero, Pauline Caraux-Paz, Chiara Simona Cardellino, Filipa Cardoso, Filipe Cardoso, Nelson Cardoso, Sofia Cardoso, Simone Carelli, Nicolas Carlier, Thierry Carmoi, Gayle Carney, Chloe Carpenter, Inês Carqueja, Marie-Christine Carret, François Martin Carrier, Gail Carson, Maire-Laure Casanova, Mariana Cascão, José Casimiro, Bailey Cassandra, Nidyanara Castanheira, Guylaine Castor-Alexandre, Henry Castrillón, Ivo Castro, Ana Catarino, François-Xavier Catherine, Paolo Cattaneo, Roberta Cavalin, Giulio Giovanni Cavalli, Alexandros Cavayas, Minerva Cervantes-Gonzalez, Anissa Chair, Catherine Chakveatze, Adrienne Chan, Meera Chand, Christelle Chantalat Auger, Jean-Marc Chapplain, Julie Chas, Mobin Chaudary, Jonathan Samuel Chávez Iñiguez, Anjellica Chen, Yih-Sharng Chen, Matthew Pellan Cheng, Antoine Cheret, Thibault Chiarabini, Julian Chica, Suresh Kumar Chidambaram, Leong Chin Tho, Catherine Chirouze, Davide Chiumello, Hwa Jin Cho, Bernard Cholley, Marie-Charlotte Chopin, Ting Soo Chow, Hiu Jian Chua, Jonathan Chua, José Pedro Cidade, José Miguel Cisneros Herreros, Barbara Wanjiru Citarella, Anna Ciullo, Jennifer Clarke, Sara Clohisey, Perren J. Cobb, Necsoi Coca, Cassidy Codan, Caitriona Cody, Alexandra Coelho, Gwenhaël Colin, Michael Collins, Sebastiano Maria Colombo, Pamela Combs, Marie Connor, Anne Conrad, Sofía Contreras, Elaine Conway, Graham S. Cooke, Mary Copland, Hugues Cordel, Amanda Corley, Sarah Cormican, Sabine Cornelis, Alexander Daniel Cornet, Arianne Joy Corpuz, Andrea Cortegiani, Grégory Corvaisier, Camille Couffignal, Sandrine Couffin-Cadiergues, Roxane Courtois, Stéphanie Cousse, Sabine Croonen, Gloria Crowl, Jonathan Crump, Claudina Cruz, Claudina Cruz, Juan Luis Cruz Berm, Jaime Cruz Rojo, Marc Csete, Alberto Cucino, Caroline Cullen, Gerard Curley, Elodie Curlier, Paula Custodio, Ana da Silva Filipe, Charlene Da Silveira, Al-Awwab Dabaliz, Andrew Dagens, Heidi Dalton, Jo Dalton, Nick Daneman, Corinne Daniel, Emmanuelle A Dankwa, Jorge Dantas, Frédérick D'Aragon, Etienne De Montmollin, Rafael Freitas de Oliveira França, Rosanna De Rosa, Thushan de Silva, Peter de Vries, David Dean, Alexa Debard, Marie-Pierre Debray, Nathalie DeCastro, William Dechert, Lauren Deconninck, Romain Decours, Eve Defous, Isabelle Delacroix, Eric Delaveuve, Karen Delavigne, Nathalie M. Delfos, Andrea Dell'Amore, Christelle Delmas, Pierre Delobel, Corine Delsing, Elisa Demonchy, Emmanuelle Denis, Dominique Deplanque, Pieter Depuydt, Mehul Desai, Diane Descamps, Mathilde Desvallée, Santi Dewayanti, Alpha Diallo, Sylvain Diamantis, André Dias, Juan Jose Diaz, Rodrigo Diaz, Kévin Didier, Jean-Luc Diehl, Wim Dieperink, Jérôme Dimet, Vincent Dinot, Fara Diop, Alphonsine Diouf, Yael Dishon, Félix Djossou, Annemarie B. Docherty, Arjen M Dondorp, Christl A. Donnelly, Maria Donnelly, Chloe Donohue, Céline Dorival, Eric D'Ortenzio, James Joshua Douglas, Renee Douma, Nathalie Dournon, Triona Downer, Mark Downing, Tom Drake, Aoife Driscoll, Murray Dryden, Claudio Duarte Fonseca, Vincent Dubee, François Dubos, Alexandre Ducancelle, Toni Duculan, Susanne Dudman, Abhijit Duggal, Paul Dunand, Jake Dunning, Mathilde Duplaix, Emanuele Durante-Mangoni, Lucian Durham III, Bertrand Dussol, Juliette Duthoit, Xavier Duval, Anne Margarita Dyrhol-Riise, Sim Choon Ean, Marco Echeverria-Villalobos, Siobhan Egan, Mohammed El Sanharawi, Subbarao Elapavaluru, Brigitte Elharrar, Rachael Ellis, Philippine Eloy, Tarek Elshazly, Isabelle Enderle, Chan Chee Eng, Ilka Engelmann, Vincent Enouf, Olivier Epaulard, Martina Escher, Mariano Esperatti, Hélène Esperou, Marina Esposito-Farese, João Estevão, Manuel Etienne, Nadia Ettalhaoui, Anna Greti Everding, Mirjam Evers, Isabelle Fabre, Marc Fabre, Amna Faheem, Arabella Fahy, Cameron J. Fairfield, Pedro Faria, Ahmed Farooq, Nataly Farshait, Hanan Fateena, Arie Zainul Fatoni, Karine Faure, Raphaël Favory, Mohamed Fayed, Niamh Feely, Jorge Fernandes, Marília Fernandes, Susana Fernandes, François-Xavier Ferrand, Eglantine Ferrand Devouge, Joana Ferrão, Mário Ferraz, Benigno Ferreira, Sílvia Ferreira, Ricard Ferrer-Roca, Nicolas Ferriere, Céline Ficko, Claudia Figueiredo-Mello, Juan Fiorda, Thomas Flament, Clara Flateau, Tom Fletcher, Letizia Lucia Florio, Brigid Flynn, Claire Foley, Victor Fomin, Tatiana Fonseca, Simon Forsyth, Denise Foster, Giuseppe Foti, Erwan Fourn, Robert A. Fowler, Marianne Fraher, Diego Franch-Llasat, Christophe Fraser, John F Fraser, Marcela Vieira Freire, Ana Freitas Ribeiro, Caren Friedrich, Ricardo Fritz, Stéphanie Fry, Nora Fuentes, Masahiro Fukuda, Valérie Gaborieau, Rostane Gaci, Massimo Gagliardi, Jean-Charles Gagnard, Amandine Gagneux-Brunon, Sérgio Gaião, Linda Gail Skeie, Phil Gallagher, Carrol Gamble, Yasmin Gani, Arthur Garan, Rebekha Garcia, Noelia García Barrio, Esteban Garcia-Gallo, Denis Garot, Valérie Garrait, Nathalie Gault, Aisling Gavin, Anatoliy Gavrylov, Alexandre Gaymard, Johannes Gebauer, Eva Geraud, Louis Gerbaud Morlaes, Nuno Germano, Jade Ghosn, Marco Giani, Tristan Gigante, Morgane Gilg, Guillermo Giordano, Michelle Girvan, Valérie Gissot, Judy Gitahi, Daniel Glikman, Petr Glybochko, Eric Gnall, François Goehringer, Jean-Christophe Goffard, Jin Yi Goh, Jonathan Golob, Marie Gominet, Alicia Gonzalez, Isabelle Gorenne, Laure Goubert, Cécile Goujard, Tiphaine Goulenok, Margarite Grable, Edward Wilson Grandin, Pascal Granier, Giacomo Grasselli, Lorenzo Grazioli, Christopher A. Green, William Greenhalf, Segolène Greffe, Domenico Luca Grieco, Matthew Griffee, Fiona Griffiths, Albert Groenendijk, Heidi Gruner, Heidi Gruner, Yusing Gu, Jérémie Guedj, Martin Guego, Dewi Guellec, Daniela Guerreiro, Romain Guery, Anne Guillaumot, Laurent Guilleminault, Maisa Guimarães de Castro, Thomas Guimard, Marieke Haalboom, Daniel Haber, Ali Hachemi, Nadir Hadri, Olena Haidash, Saeeda Haider, Fakhir Haidri, Adam Hall, Matthew Hall, Sophie Halpin, Ansley Hamer, Rebecca Hamidfar, Terese Hammond, Lim Yuen Han, Rashan Haniffa, Kok Wei Hao, Hayley Hardwick, Ewen M. Harrison, Janet Harrison, Alan Hartman, Madiha Hashmi, Muhammad Hayat, Leanne Hays, Jan Heerman, Lars Heggelund, Ross Hendry, Martina Hennessy, Aquiles Henriquez-Trujillo, Maxime Hentzien, Fivzia Herekar, Andrew Hershey, Liv Hesstvedt, Dawn Higgins, Eibhilin Higgins, Samuel Hinton, Hiroaki Hiraiwa, Hikombo Hitoto, Antonia Ying Wai Ho, Yi Bin Ho, Alexandre Hoctin, Isabelle Hoffmann, Wei Han Hoh, Oscar Hoiting, Rebecca Holt, Jan Cato Holter, Peter Horby, Koji Hoshino, Kota Hoshino, Ikram Houas, Catherine L. Hough, Stuart Houltham, Jimmy Ming-Yang Hsu, Jean-Sébastien Hulot, Iqbal Hussain, Samreen Ijaz, Hajnal-Gabriela Illes, Patrick Imbert, Rana Imran Sikander, Hugo Inácio, Carmen Infante Dominguez, Yun Sii Ing, Elias Iosifidis, Mariachiara Ippolito, Sarah Isgett, Palliya Guruge Pramodya Ishani Ishani, Tiago Isidoro, Nadiah Ismail, Margaux Isnard, Junji Itai, Daniel Ivulich, Danielle Jaafar, Salma Jaafoura, Julien Jabot, Clare Jackson, Nina Jamieson, Pierre Jaquet, Waasila Jassat, Coline Jaud-Fischer, Stéphane Jaureguiberry, Issrah Jawad, Devachandran Jayakumar, Florence Jego, Anilawati Mat Jelani, Synne Jenum, Ruth Jimbo-Sotomayor, van der Palen Job, Ong Yiaw Joe, Ruth N. Jorge García, Cédric Joseph, Mark Joseph, Mercé Jourdain, Philippe Jouvet, Jennifer June, Anna Jung, Hanna Jung, Dafsah Juzar, Ouifiya Kafif, Florentia Kaguelidou, Neerusha Kaisbain, Thavamany Kaleesvran, Sabina Kali, Smaragdi Kalomoiri, Muhammad Aisar Ayadi Kamaluddin, Zul Amali Che Kamaruddin, Nadiah Kamarudin, Darshana Hewa Kandamby, Chris Kandel, Kong Yeow Kang, Ravi Kant, Darakhshan Kanwal, Pratap Karpayah, Christiana Kartsonaki, Daisuke Kasugai, Anant Kataria, Kevin Katz, Simreen Kaur Johal, Christy Kay, Seán Keating, Andrea Kelly, Sadie Kelly, Ryan Kennedy, Kalynn Kennon, Younes Kerroumi, Evelyne Kestelyn, Imrana Khalid, Osama Khalid, Antoine Khalil, Coralie Khan, Irfan Khan, Sushil Khanal, Michelle E Kho, Denisa Khoo, Ryan Khoo, Saye Khoo, Nasir Khoso, Khor How Kiat, Yuri Kida, Peter Kiiza, Anders Benjamin Kildal, Jae Burm Kim, Antoine Kimmoun, Alexander King, Nobuya Kitamura, Paul Klenerman, Rob Klont, Gry Kloumann Bekken, Stephen Knight, Chamira Kodippily, Sabin Koirala, Mamoru Komatsu, Caroline Kosgei, Arsène Kpangon, Karolina Krawczyk, Sudhir Krishnan, Vinothini Krishnan, Oksana Kruglova, Ashok Kumar, Deepali Kumar, Ganesh Kumar, Pavan Kumar Vecham, Ethan Kurtzman, Demetrios Kutsogiannis, Galyna Kutsyna, Konstantinos Kyriakoulis, Marie Lachatre, Marie Lacoste, John G. Laffey, Marie Lagrange, Fabrice Laine, Olivier Lairez, Antonio Lalueza, Marc Lambert, François Lamontagne, Marie Langelot-Richard, Vincent Langlois, Eka Yudha Lantang, Marina Lanza, Cédric Laouénan, Samira Laribi, Delphine Lariviere, Stéphane Lasry, Naveed Latif, Odile Launay, Didier Laureillard, Yoan Lavie-Badie, Andy Law, Cassie Lawrence, Teresa Lawrence, Minh Le, Clément Le Bihan, Cyril Le Bris, Georges Le Falher, Lucie Le Fevre, Quentin Le Hingrat, Marion Le Maréchal, Soizic Le Mestre, Gwenaël Le Moal, Vincent Le Moing, Hervé Le Nagard, Paul Le Turnier, Ema Leal, Marta Leal Santos, Biing Horng Lee, Heng Gee Lee, James Lee, Su Hwan Lee, Todd C. Lee, Yi Lin Lee, Gary Leeming, Bénédicte Lefebvre, Laurent Lefebvre, Benjamin Lefevre, Sylvie LeGac, Jean-Daniel Lelievre, François Lellouche, Adrien Lemaignen, Véronique Lemee, Anthony Lemeur, Gretchen Lemmink, Ha Sha Lene, Marc Leone, Michela Leone, Quentin Lepiller, François-Xavier Lescure, Olivier Lesens, Mathieu Lesouhaitier, Bruno Levy, Yves Levy, Claire Levy-Marchal, Erwan L'Her, Gianluigi Li Bassi, Janet Liang, Ali Liaquat, Geoffrey Liegeon, Kah Chuan Lim, Wei Shen Lim, Chantre Lima, Bruno Lina, Lim Lina, Andreas Lind, Guillaume Lingas, Sylvie Lion-Daolio, Keibun Liu, Marine Livrozet, Patricia Lizotte, Antonio Loforte, Navy Lolong, Leong Chee Loon, Diogo Lopes, Dalia Lopez-Colon, Anthony L. Loschner, Paul Loubet, Bouchra Loufti, Guillame Louis, Silvia Lourenco, Lee Lee Low, Marije Lowik, Jia Shyi Loy, Jean Christophe Lucet, Carlos Lumbreras Bermejo, Carlos M. Luna, Liem Luong, Nestor Luque, Dominique Luton, Nilar Lwin, Ruth Lyons, Olavi Maasikas, Oryane Mabiala, Samual MacDonald, Moïse Machado, Gabriel Macheda, Guillermo Maestro de la Calle, Rafael Mahieu, Sophie Mahy, Lars S. Maier, Mylène Maillet, Thomas Maitre, Maximilian Malfertheiner, Nadia Malik, Fernando Maltez, Denis Malvy, Victoria Manda, Laurent Mandelbrot, Frank Manetta, Julie Mankikian, Edmund Manning, Aldric Manuel, Ceila Maria Sant`Ana Malaque, Flávio Marino, Patrick Mark , Samuel Markowicz, Charbel Maroun Eid, Ana Marques, Catherine Marquis, Brian Marsh, Laura Marsh, John Marshall, Celina Turchi Martelli, Emily Martin, Alejandro Martín Quirós , Guillaume Martin-Blondel, Alessandra Martinelli, Ignacio Martin-Loeches, Martin Martinot, Ana Martins, João Martins, Caroline Martins Rego, Gennaro Martucci, Olga Martynenko, Eva Miranda Marwali, Marsilla Marzukie, David Maslove, Sabina Mason, Sobia Masood, Sobia Masood, Moshe Matan, Meghena Mathew, Daniel Mathieu, Mathieu Mattei, Romans Matulevics, Laurence Maulin, Javier Maynar, Thierry Mazzoni, Natalie Mc Evoy, Colin McArthur, Aine McCarthy, Anne McCarthy, Colin McCloskey, Rachael McConnochie, Sherry McDermott, Sarah E. McDonald, Samuel McElwee, Allison McGeer, Chris McKay, Johnny McKeown, Kenneth A. McLean, Bairbre McNicholas, Edel Meaney, Cécile Mear-Passard, Maggie Mechlin, Omar Mehkri, Ferruccio Mele, France Mentré, Alexander J. Mentzer, Emmanuelle Mercier, Noémie Mercier, Antoine Merckx, Mayka Mergeay-Fabre, Blake Mergler, Laura Merson, António Mesquita, Agnès Meybeck, Dan Meyer, Alison M. Meynert, Vanina Meysonnier, Amina Meziane, Mehdi Mezidi, Céline Michelanglei, Isabelle Michelet, Efstathia Mihelis, Vladislav Mihnovit, Hugo Miranda-Maldonado, Nor Arisah Misnan, Nik Nur Eliza Mohamed, Tahira Jamal Mohamed, Asma Moin, David Molina, Elena Molinos, Mary Mone, Agostinho Monteiro, Claudia Montes, Giorgia Montrucchio, Sarah Moore, Shona C. Moore, Lina Morales Cely, Lucia Moro, Catherine Motherway, Ana Motos, Hugo Mouquet, Clara Mouton Perrot, Julien Moyet, Caroline Mudara, Aisha Kalsoom Mufti, Ng Yong Muh, Dzawani Muhamad, Jimmy Mullaert, Fredrik Muller, Karl Erik Müller, Daniel Munblit, Syed Muneeb, Marlène Murris, Srinivas Murthy, Gugapriyaa Muyandy, Dimitra Melia Myrodia, Namra N, Dave Nagpal, Alex Nagrebetsky, Mangala Narasimhan, Rashid Nasim Khan, Nadège Neant, Nikita A Nekliudov, Raul Neto, Emily Neumann, Pauline Yeung Ng, Wing Yiu Ng, Anthony Nghi, Duc Nguyen, Orna Ni Choileain, Alistair Nichol, Prompak Nitayavardhana, Stephanie Nonas, Nurul Amani Mohd Noordin, Marion Noret, Nurul Faten Izzati Norharizam, Lisa Norman, Alessandra Notari, Mahdad Noursadeghi, Saad Nseir, Jose I Nunez, Elsa Nyamankolly, Giovanna Occhipinti, Takayuki Ogura, Tak-Hyuk Oh, Shinichiro Ohshimo, João Oliveira, Larissa Oliveira, Piero L. Olliaro, Conar O'Neil, David S.Y. Ong, Jee Yan Ong, Wilna Oosthuyzen, Anne Opavsky, Peter Openshaw, Saijad Orakzai, Claudia Milena Orozco-Chamorro, Jamel Ortoleva, Javier Osatnik, Siti Zubaidah Othman, Nadia Ouamara, Rachida Ouissa, Eric Oziol, H M Upulee Pabasara, Maïder Pagadoy, Justine Pages, Amanda Palacios, Mario Palacios, Massimo Palmarini, Giovanna Panarello, Prasan Kumar Panda, Hem Paneru, Lai Hui Pang, Mauro Panigada, Nathalie Pansu, Aurélie Papadopoulos, Rachael Parke, Briseida Parra, Taha Pasha, Jérémie Pasquier, Bruno Pastene, Ana Cecilia Pastor Ludeña, Fabian Patauner, Junaid Patel, Mohan Dass Pathmanathan, Patricia Patricio, Juliette Patrier, Lisa Patterson, Rajyabardhan Pattnaik, Christelle Paul, Mical Paul, Jorge Paulos, William A. Paxton, Jean-François Payen, Kalaiarasu Peariasamy, Miguel Pedrera Jiménez, Giles J. Peek, Florent Peelman, Nathan Peiffer-Smadja, Vincent Peigne, Mare Pejkovska, Paolo Pelosi, Ithan D. Peltan, Rui Pereira, Daniel Perez, Luis Periel, Thomas Perpoint, Antonio Pesenti, Vincent Pestre, Lenka Petrou, Ventzislava Petrov-Sanchez, Frank Olav Pettersen, Gilles Peytavin, Scott Pharand, Michael Piagnerelli, Walter Picard, Olivier Picone, Carola Pierobon, Djura Piersma, Carlos Pimentel, Raquel Pinto, Catarina Pires, Isabelle Pironneau, Lionel Piroth, Riinu Pius, Simone Piva, Laurent Plantier, Daniel Plotkin, Hon Shen Png, Julien Poissy, Ryadh Pokeerbux, Sergio Poli, Georgios Pollakis, Diane Ponscarme, Diego Bastos Porto, Andra-Maris Post, Douwe F. Postma, Pedro Póvoa, Diana Póvoas, Jeff Powis, Sofia Prapa, Sébastien Preau, Christian Prebensen, Jean-Charles Preiser, Anton Prinssen, Mark G. Pritchard, Gamage Dona Dilanthi Priyadarshani, Lucia Proença, Oriane Puéchal, Bambang Pujo Semedi, Gregory Purcell, Luisa Quesada, Víctor Quirós González, Else Quist-Paulsen, Mohammed Quraishi, Christian Rabaud, Ebenezer Rabindrarajan, Aldo Rafael, Marie Rafiq, Ahmad Kashfi Haji Ab Rahman, Rozanah Abd Rahman, Arsalan Rahutullah, Fernando Rainieri, Giri Shan Rajahram, Nagarajan Ramakrishnan, Ahmad Afiq Ramli, Blandine Rammaert, Grazielle Viana Ramos, Asim Rana, Christophe Rapp, Aasiyah Rashan, Thalha Rashan, Ghulam Rasheed, Menaldi Rasmin, Indrek Rätsep, Tharmini Ravi, Ali Raza, Andre Real, Stanislas Rebaudet, Sarah Redl, Brenda Reeve, Ali Rehan, Attaur Rehman, Liadain Reid, Dag Henrik Reikvam, Renato Reis, Jordi Rello, Martine Remy, Hongru Ren, Liliana Resende, Anne-Sophie Resseguier, Matthieu Revest, Oleksa Rewa, Luis Felipe Reyes, Tiago Reyes, Maria Ines Ribeiro, David Richardson, David Richardson, Denise Richardson, Laurent Richier, Siti Nurul Atikah Ahmad Ridzuan, Jordi Riera, Ana L Rios, Asgar Rishu, Patrick Rispal, Karine Risso, Maria Angelica Rivera Nuñez, Nicholas Rizer, Chiara Robba, André Roberto, Stephanie Roberts, David L. Robertson, Olivier Robineau, Ferran Roche-Campo, Paola Rodari, Simão Rodeia, Bernhard Roessler, Pierre-Marie Roger, Emmanuel Roilideseira, Amanda Rojek, Juliette Romaru, Roberto Roncon-Albuquerque Jr, Mélanie Roriz, Manuel Rosa-Calatrava, Michael Rose, Dorothea Rosenberger, Nurul Hidayah Mohammad Roslan, Andrea Rossanese, Matteo Rossetti, Bénédicte Rossignol, Patrick Rossignol, Stella Rousset, Carine Roy, Benoît Roze, Clark D. Russell, Steffi Ryckaert, Aleksander Rygh Holten, Isabela Saba, Sairah Sadaf, Musharaf Sadat, Valla Sahraei, Maximilien Saint-Gilles, Pranya Sakiyalak, Nawal Salahuddin, Leonardo Salazar, Jodat Saleem, Gabriele Sales, Stéphane Sallaberry, Charlotte Salmon Gandonniere, Hélène Salvator, Olivier Sanchez, Angel Sanchez-Miralles, Vanessa Sancho-Shimizu, Gyan Sandhu, Pierre-François Sandrine, Oana Sandulescu, Marlene Santos, Shirley Sarfo-Mensah, Bruno Sarmento Banheiro, Iam Claire E. Sarmiento, Benjamine Sarton, Sree Satyapriya, Rumaisah Satyawati, Egle Saviciute, Parthena Savvidou, Yen Tsen Saw, Justin Schaffer, Tjard Schermer, Arnaud Scherpereel, Marion Schneider, Stephan Schroll, Michael Schwameis, Janet T. Scott, James Scott-Brown, Nicholas Sedillot, Tamara Seitz, Mageswari Selvarajoo, Caroline Semaille, Malcolm G. Semple, Rasidah Bt Senian, Eric Senneville, Claudia Sepulveda, Filipa Sequeira, Tânia Sequeira, Ary Serpa Neto, Pablo Serrano Balazote, Ellen Shadowitz, Syamin Asyraf Shahidan, Mohammad Shamsah, Pratima Sharma, Catherine A. Shaw, Victoria Shaw, Ashraf Sheharyar, Haixia Shi, Mohiuddin Shiekh, Nobuaki Shime, Hiroaki Shimizu, Keiki Shimizu, Sally Shrapnel, Hoi Ping Shum, Nassima Si Mohammed, Ng Yong Siang, Jeanne Sibiude, Atif Siddiqui, Louise Sigfrid, Piret Sillaots, Catarina Silva, Maria Joao Silva, Rogério Silva, Benedict Sim Lim Heng, Wai Ching Sin, Budha Charan Singh, Pompini Agustina Sitompul, Karisha Sivam, Vegard Skogen, Sue Smith, Benjamin Smood, Michelle Smyth, Morgane Snacken, Dominic So, Tze Vee Soh, Monserrat Solis, Joshua Solomon, Tom Solomon, Emily Somers, Agnès Sommet, Myung Jin Song, Rima Song, Tae Song, Azlan Mat Soom, Alberto Sotto, Edouard Soum, Ana Chora Sousa, Marta Sousa, Maria Sousa Uva, Vicente Souza-Dantas, Alexandra Sperry, Elisabetta Spinuzza, B. P. Sanka Ruwan Sri Darshana, Shiranee Sriskandan, Sarah Stabler, Thomas Staudinger, Stephanie-Susanne Stecher, Trude Steinsvik, Ymkje Stienstra, Birgitte Stiksrud, Adrian Streinu-Cercel, Anca Streinu-Cercel, Samantha Strudwick, Ami Stuart, David Stuart, Gabriel Suen, Jacky Y. Suen, Michael K Sullivan, Asfia Sultana, Charlotte Summers, Deepashankari Suppiah, Magdalena Surovcová, Andrey A Svistunov, Sarah Syahrin, Konstantinos Syrigos, Jaques Sztajnbok, Konstanty Szuldrzynski, Shirin Tabrizi, Fabio S. Taccone, Lysa Tagherset, Shahdattul Mawarni Taib, Sara Taleb, Jelmer Talsma, Maria Lawrensia Tampubolon, Kim Keat Tan, Le Van Tan, Yan Chyi Tan, Hiroyuki Tanaka, Taku Tanaka, Hayato Taniguchi, Hussain Tanveer, Huda Taqdees, Arshad Taqi, Coralie Tardivon, Pierre Tattevin, M Azhari Taufik, Richard S. Tedder, Sofia Tejada, Tze Yuan Tee, João Teixeira, Marie-Capucine Tellier, Sze Kye Teoh, Vanessa Teotonio, François Téoulé, Pleun Terpstra, Olivier Terrier, Nicolas Terzi, Hubert Tessier-Grenier, Adrian Tey, Alif Adlan Mohd Thabit, Zhang Duan Tham, Suvintheran Thangavelu, Vincent Thibault, Simon-Djamel Thiberville, Benoît Thill, Jananee Thirumanickam, Shaun Thompson, David Thomson, Emma C. Thomson, Surain Raaj Thanga Thurai, Duong Bich Thuy, Ryan S. Thwaites, Vadim Tieroshyn, Peter S Timashev, Jean-François Timsit, Bharath Kumar Tirupakuzhi Vijayaraghavan, Noémie Tissot, Jordan Zhien Yang Toh, Maria Toki, Timo Tolppa, Kristian Tonby, Sia Loong Tonnii, Antoni Torres, Margarida Torres, Rosario Maria Torres Santos-Olmo, Hernando Torres-Zevallos, Tony Trapani, Théo Treoux, Huynh Trung Trieu, Swagata Tripathy, Cécile Tromeur, Ioannis Trontzas, Tiffany Trouillon, Jeanne Truong, Christelle Tual, Sarah Tubiana, Helen Tuite, Jean-Marie Turmel, Lance C.W. Turtle, Anders Tveita, Pawel Twardowski, PG Ishara Udayanga, Andrew Udy, Roman Ullrich, Alberto Uribe, Asad Usman, Tim Uyeki, Luís Val-Flores, Amélie Valran, Stijn Van de Velde, Marcel van den Berge, Machteld Van der Feltz, Paul van der Valk, Nicky Van Der Vekens, Peter Van der Voort, Sylvie Van Der Werf, Laura van Gulik, Jarne Van Hattem, Steven van Lelyveld, Carolien van Netten, Gitte Van Twillert, Ilonka van Veen, Noémie Vanel, Henk Vanoverschelde, Michael Varrone, Shoban Raj Vasudayan, Charline Vauchy, Shaminee Veeran, Aurélie Veislinger, Sara Ventura, Annelies Verbon, José Ernesto Vidal, César Vieira, Deepak Vijayan, Joy Ann Villanueva, Andrea Villoldo, Nguyen Van Vinh Chau, Benoit Visseaux, Chiara Vitiello, Harald Vonkeman, Fanny Vuotto, Suhaila Abdul Wahab, Nadirah Abdul Wahid, Chih-Hsien Wang, Steve Webb, Jia Wei, Tan Pei Wen, Sanne Wesselius, Murray Wham, Bryan Whelan, Nicole White, Paul Henri Wicky, Aurélie Wiedemann, Surya Otto Wijaya, Keith Wille, Suzette Willems, Virginie Williams, Evert-Jan Wils, Timo Wolf, Calvin Wong, Teck Fung Wong, Xin Ci Wong, Yew Sing Wong, Gan Ee Xian, Lim Saio Xian, Kuan Pei Xuan, Ioannis Xynogalas, Sophie Yacoub, Siti Rohani Binti Mohd Yakop, Masaki Yamazaki, Yazdan Yazdanpanah, Nicholas Yee Liang Hing, Cécile Yelnik, Chian Hui Yeoh, Stephanie Yerkovich, Toshiki Yokoyama, Hodane Yonis, Akram Zaaqoq, Marion Zabbe, Masliza Zahid, Maram Zahran, Nor Zaila Binti Zaidan, Maria Zambon, Miguel Zambrano, Alberto Zanella, Nurul Zaynah, Hiba Zayyad, Alexander Zoufaly, David Zucman, The Western Australian COVID-19 Research Response, Mazankowski Heart Institute.
