# Supplementary material for: Use of an extended KDIGO definition to diagnose acute kidney injury in patients with COVID-19: A multinational study using the ISARIC–WHO clinical characterisation protocol
Source: PLoS Med. 2022 Apr 20;19(4):e1003969. doi: 10.1371/journal.pmed.1003969 (PMC9067700; doi:10.1371/journal.pmed.1003969)
Supplement: Attachment — Submitted filename: Reply letter_PLOS Med_Revisions_FINAL.docx [file pmed.1003969.s010.docx]

Reply letter to PLOS Medicine Editors and Reviewers

Dear Editor,

Thank you for the extensive feedback provided for our paper “Acute Kidney Injury in Patients with COVID-19 using an Extended KDIGO Definition”. We very much appreciate the time and effort put forth by reviewers and editors and believe all the suggested changes have greatly improved the paper. As per the suggestions we have now included the comparisons between KDIGO, eKDIGO and deKDIGO groups as well as a survival analysis and a logistic regression.

Sincerely,

Marina Wainstein and Sally Shrapnel

(On behalf of all authors)

Editor

1. *Please revise your title according to PLOS Medicine's style. Your title must be nondeclarative and not a question. It should begin with main concept if possible. "Effect of" should be used only if causality can be inferred, i.e., for an RCT. Please place the study design (e.g. a "a cohort study") in the subtitle (ie, after a colon).*

**The title and subtitle have been changed to the following:**

**Title: Use of an Extended KDIGO Definition to diagnose Acute Kidney Injury in Patients with COVID-19**

**Subtitle: A prospective, multinational, observational study of the ISARIC cohort.**

2. *Please structure your abstract using the PLOS Medicine headings (Background, Methods and Findings, Conclusions). Please combine the abstract Methods and Results sections into one section, “Methods and findings”.*

**This has been done according to PLOS Medicine guidelines**

3. *Abstract Background: The final sentence should clearly state the study question.*

**The following has been added to the abstract background:**

***“We hypothesized that an extended KDIGO definition, adapted from the ISN 0by25 studies, would identify more cases of AKI in patients with COVID-19. We also hypothesized that the additional cases identified using this extended criterion may correspond to CA-AKI and be associated with similarly poor outcomes as those shown in previous studies of AKI in COVID-19.” (lines 6-10)***

4. *Abstract Methods and Findings:
   * Please include the study design, years during which the study took place, length of follow up, and main outcome measures.
   * Please quantify the main results (with 95% CIs and p values).
   * Please include the important dependent variables that are adjusted for in the analyses.
   * In the last sentence of the Abstract Methods and Findings section, please describe the main limitation(s) of the study's methodology.*

**These changes have been made accordingly. (lines 2 – 43)**

5. *At this stage, we ask that you include a short, non-technical Author Summary of your research to make findings accessible to a wide audience that includes both scientists and non-scientists.*

**This has been done and added after the abstract**

6. *Please review the submission guidelines and confirm that your financial disclosure only contains the relevant information.*

**This has been done and the financial disclosure section has been updated.**

7. *Please review the submission guidelines and confirm that your competing interests statement only contains the relevant information.*

**This has been done and the competing interests statement updated**

8. *The Data Availability Statement (DAS) requires revision. For the small subset of sites that have not agreed to pooled data sharing, the point of contact for inquiries cannot be a study author.*

**The Data Availability Statement (DAS) has been revised and modified to address the above comment in the online submission.**

9. *Citations should be in square brackets, and preceding punctuation.*

**All citations have been revised accordingly**

10. *Please define the length of follow up (eg, in mean, SD, and range).*

**In our study, data was collected and analysed during the length of a patient’s hospital admission between February 15^th^ 2020 and February 1^st^ 2021. The median length of admission was 12 days (IQR 7,20 days). This has been added to the Methods section (lines 187-188) and Results section (line 244)**

11. *When a p value is given, please specify the statistical test used to determine it.*

**All tests used to calculate p-values have been listed in the statistical analysis section of the methods section. (lines 213 – 221)**

12. *Please define the abbreviations in Figure 1.*

**This has been done (see Fig 1).**

13. *Please provide the unadjusted comparisons as well as the adjusted comparisons in Table 3.*

**The unadjusted comparisons between AKI and in-hospital death among eKDIGO, deKDIGO and KDIGO have been provided in the Outcome section of Tables 2, 3 and 4. The adjusted comparison between eKDIGO AKI and death is shown in the logistic regression in table 5.**

14. *Please specify the variables controlled for in Table 3.*

**This has been added to the statistical analysis section (lines 223 – 229)**

15. *Please provide the name(s) of the institutional review board(s) that provided ethical approval (this can be provided in the supplementary materials if required).*

*You*

**The full statement can be found in S1 statement and a shortened version in Methods lines 130 - 132**

16. *Please specify whether informed consent was written or oral.*

**This has been added in Methods lines 127 - 129:**

***“Protocol and consent forms are available at*** [***https://isaric.net/ccp/***](https://isaric.net/ccp/)***. While written consent was obtained in most cases, for some sites the local regulators and ethics committees approved oral consent, or waiver of consent, in the context of the pandemic.”***

**Unfortunately, we are unable to access an up-to-date list of which centres required written consent and which centres permitted oral consent or a waiver of consent.**

17. *Please ensure that the study is reported according to the STROBE guideline, and include the completed STROBE checklist as Supporting Information.*

**It has been added to supplementary material (S5 Table)**

18. *Please add the following statement, or similar, to the Methods: "This study is reported as per the Strengthening the Reporting of Observational Studies in Epidemiology (STROBE) guideline (S1 checklist).*

**This has been added to the Methods section, lines 237-238**

19. *Did your study have a prospective analysis plan? Please state this (either way) early in the Methods section.*
    a. *If a prospective analysis plan (from your funding proposal, IRB or other ethics committee submission, study protocol, or other planning document written before analyzing the data) was used in designing the study, please include the relevant prospectively written document with your revised manuscript as a Supporting Information file to be published alongside your study, and cite it in the Methods section. A legend for this file should be included at the end of your manuscript.*

**The prospective analysis plan has been described in the methods section (lines 133-136) and cited in the references [12] with a link to the online pdf file**

b. *In either case, changes in the analysis-- including those made in response to peer review comments-- should be identified as such in the Methods section of the paper, with rationale.*

**This is explained in the methods section, lines 133-136**

20. *In the first sentence of the concluding paragraph in your discussion, please temper claims of primacy by stating, "to our knowledge" or something similar.*

**This statement has been tempered.**

Academic Editor

21. *Need to provide in the main manuscript the comparison of the outcomes between that part of eKDIGO criteria which otherwise would be missed by standard KDIGO criteria;*

**Please refer to the outcomes section in tables 3 and 4.**

22. *Need to provide a table showing differences between AKI diagnosed by deKDIGO and KDIGO criteria, and deKDIGO and no AKI;*

**These have been added to the results section: Tables 3 and 4. We have also added the rationale for the inclusion of RRT patients only in the KDIGO group to caution the reader when looking at side-by-side comparisons of these groups (172 – 175):**

***“Information on the timing of acute RRT was not always available so it was not possible to determine whether patients receiving RRT fell into the KDIGO or deKDIGO portions of the eKDIGO group. Given the low likelihood that a patient with a falling creatinine would be given acute RRT, all RRT patients were categorized within the KDIGO group as stage 3 AKI.”***

23. *Unclear whether there were any readmission, and how the authors treated readmission in the analysis;*

**Thank you for identifying this important limitation. We had limited data on readmissions and therefore could not reliably identify them (Methods line 210). The following has been added to the discussion section (345 – 347):**

***“While the inability to exclude readmitted patients may have introduced a degree of survival bias, the fact that readmission rates of less than 3% are seen in other large studies, suggests that the effect of this bias is likely to be relatively small” [1-3]***

24. *Need to explain why in the present manuscript mortality may be lower than that reported in a recent UK study, since the first phase of COVID-19 pandemic was associated with high incidence of AKI and high mortality, especially in Europe;*

**When using the KDIGO AKI criteria, which is what most studies have used, our mortality rate among AKI patients falls within the 34% and 50% reported in previous studies [1, 3-6]. Note that the 60.5% mortality in the Kokle et al paper is at the upper end of incidence. We have added the following statement to the discussion:**

***“The mortality rate of 50% among KDIGO-diagnosed AKI patients falls within the range (34 to 50%) reported in previous studies using the same AKI definition [1-5].***” **(lines 343 – 344)**

|  | **F/U period** | **Death No AKI** | **Death AKI pts (KDIGO)** |
| --- | --- | --- | --- |
| **Wainstein et al** | Feb 2020 to Feb 2021 | 19% | KDIGO 50%, eKDIGO 38%, deKDIGO 25% |
| **Bowe et al. (US 5000 +)** | Feb 2020 to July 2020 | 8% | 34% |
| **Sullivan et al (85687 pts)** | Jan 2020 to Dec 2020 | Not reported | 40% |
| **Kohle PLOS (4759 pts)** | March 2020 to May 2020 | 27.4% | 60.5% |
| **Chan et al JASN (4000 pts)** | Feb 2020 to May 2020 | 8% | 50% |
| **Hirsch et al (5449 pts)** | March 1 – April 2020 | 5.6% | 35% |
| **Ng et al (9657 pts)** | March 1 – June 4th | 7.3% | 46% |

25. *Need to explain in more depth the reasoning behind a decrease in sCr indicating AKI;*

**The rationale behind using a commensurate decrease in sCr to identify AKI is to ensure patients that have developed AKI in the community and experience some recovery of kidney function during the hospital stay are not missed (as they are when using the existing KDIGO AKI criteria). This has shown to be particularly relevant with CA-AKI in low-income settings [6]. Of note, there are few existing studies exploring a decrease in sCr as a marker of kidney injury and consequently this has been highlighted as one of the research priorities in the recent KDIGO controversies in AKI report [7]. While other papers have indicated the need to revise various aspects of the KDIGO criteria [8, 9] , aside from the 0by25 studies, a decrease in sCr as a marker of AKI has only been explored in infants and neonates. Ours is one of the first studies to pioneer this definition and explore its merits and challenges. We hope that our results will inspire other groups to validate eKDIGO across a variety of settings.**

**Excerpts of the above discussion have been added to the introduction (lines 97 – 102)**

***“The integration of this additional criteria to identify kidney injury has also been highlighted as one of the research priorities in the recent KDIGO report on controversies in AKI [7]. While other papers have indicated the need to revise various aspects of the KDIGO criteria, aside from the 0by25 studies, a decrease in serum creatinine as a marker of AKI has only been explored in infants and neonates, prompting a need for further research in this area [8, 9].”***

26. *Need to clarify the criteria contributing to the number of sCr taken for each individual, and discuss the possible bias of this criteria;*

**The criteria contributing to the number of sCr taken for each individual is now mentioned in the Methods and Discussion section:**

***“Collection of serum creatinine measurements across all sites was not time-standardized and the frequency of collection was left to the discretion of each site.”* (lines 123-124)**

***“The lack of a time-standardized collection of sCr across all sites also represents a limitation of the study. Patients having more frequent sCr collections may represent a population with more severe illness in whom AKI would be more readily detected, therefore affecting the overall AKI incidence rates and potentially generating a negative survival bias. Nevertheless, it is reassuring that the number of AKI cases are a small proportion of the total sCr collected on any given day (<18%) (S2 Fig), suggesting that the bias introduced by ad hoc sampling was low.***

***The lack of standardization in sCr collection may have also affected the reporting of time to peak AKI, the magnitude of peak AKI reached in each individual patient and, in those experiencing both a rise and fall in sCr during their admission, whether AKI was captured during the former phase (KDIGO) or the latter (eKDIGO).”* (lines 394 – 403)**

27. *Concern about the fact that the number of sCr measurements/tests taken would appear to affect the probability that a patient would meet the eKDIGO criteria;*

**We have mentioned this limitation in the discussion section:**

**“*The lack of standardization in sCr collection may have also affected the reporting of time to peak AKI, the magnitude of peak AKI reached in each individual patient and, in those experiencing both a rise and fall in sCr during their admission, whether AKI was captured during the former phase (KDIGO) or the latter (eKDIGO).”* (lines 400 – 403)**

28. *Need to clarify and present the typical sCr measurement/test schedules that would also be relevant to the presentation for peak AKI stage;*

**As sCr sampling was done in an ad hoc fashion following regular clinical care, we could not obtain this information. As per point 27, we have added a sentence in the discussion highlighting this as a limitation towards the timing and stage of AKI diagnosed.**

29. *Need to add for adjustment the age as possible confounder (Table 3);*

**This has been added to the logistic regression (Table 5)**

30. *Need to stratify the analysis by age for validation of the findings;*

**This has been done in the logistic regression using three age brackets: 18-64, 65-84 and 85+**

31. *Need to clarify how the confounders (Table 3) were chosen;*

**The rationale for how confounders were shown has been added to Methods (lines 223 – 229):**

***“Adjustments were made for factors indicating disease severity such as: admission to ICU; need for mechanical ventilation; corticosteroid and antifungal treatment; complicating factors such as bacterial pneumonia, cardiac arrest, coagulation disorders and rhabdomyolysis. Adjustment was also made for factors known to increase the susceptibility to AKI such as age, sex, diabetes, chronic cardiac disease, chronic pulmonary disease, chronic kidney disease (CKD), hypertension, obesity, and use of renin– angiotensin system blockers (RAS blockers) before admission [10].”***

32. *Provide a brief sensitivity analysis for the period before vaccination become available;*

**While we agree that this would be interesting to explore, we feel this is beyond the scope of this current paper. Vaccination introduction varies significantly across our population, making a pre- and post-vaccination analysis difficult.**

33. *Need to stratify analyses by country development;*

**Unfortunately, our final analysis cohort was comprised of a small proportion of patients from low and low middle income countries (4037 patients, 5% of total). We would therefore be concerned about drawing reliable conclusions about patients from this group, beyond AKI incidence rates (which we have presented in tables 3, 4 and 5). A thorough statistical analysis of the effect of country development across all our analyses, given the substantial class imbalance, would be a significant undertaking and is beyond the scope of this paper.**

34. *Discuss as a study limitation the fact that the involvement of patient without laboratory-confirmed SARS-CoV-2 infection, but with a clinical diagnosis of COVID-19, which relied on symptoms that could be ascribed to other infectious diseases, may have resulted in the inclusion of participants who were hospitalized for non-COVID-19-related conditions;*

**This has been added to the discussion (lines 383 - 389)**

***“In this context, it should be noted that approximately 20% of the analysis cohort had a diagnosis of COVID-19 made on clinical grounds, most likely due to testing shortages and high resource demands during surge phases of the pandemic. While this may have resulted in the potential inclusion of patients with other respiratory illnesses, given that other common respiratory illnesses were notably less prevalent during the pandemic (citation CDC/Australia), it is plausible that a significant proportion of these clinically diagnosed patients did in fact have COVID-19.”***

35. *Need to add in the logistic Generalized Additive Model, several covariates which may have affected the risk of in-hospital mortality, such as ethnicity and co-morbidities (e.g. chronic kidney disease);*

**Following the editor and reviewers comments we have added several new covariates to the mortality analysis including age, comorbidities (chronic kidney disease, type 1 and 2 diabetes, obesity, hypertension, chronic pulmonary disease, chronic cardiac disease) and use of ACE/ARBs.**

**While we agree that inclusion of ethnicity in the analysis would be very valuable given the association of increased mortality among patients of black race with AKI, unfortunately ethnicity was not reliably or consistently reported in our population. We believe this probably resulted from having such a culturally and ethnically diverse population which could not easily be captured in the traditional, broad ethnic categories presented in the CRF forms.**

36. *Concern about the conclusions regarding the generalizability of the study findings, since only about 5.3% of the overall patient population was from low- and low/middle-income countries;*

**This statement has been tempered in the conclusion section.**

***“Our population is, as far as we know, the largest and only multinational cohort of patients with COVID-19 from all income country levels.” (lines 414- 415)***

37. *Need to provide the proportion of patients who achieved recovery of kidney function by the time of hospital discharge among surviving patients who developed AKI, according to the traditional and the extended KDIGO definitions.*

**Unfortunately, this analysis cannot be done as only a small proportion of patients had sCr measurements done close to discharge: 30% (16927) of patients had a serum creatinine collected on the day of discharge, 12% had a sCr one day before discharge, 8% 2 days before D/C, 6% 3 days before %, 4% 4 days before D/C).**

Reviewer #1:

*This is a very interesting study using eKDIGO criteria for diagnosing AKI. The clinical features and outcomes of patients diagnosed by falling creatinine in subsequent 7 days is important.*

38. *Comparison of the outcomes between that part of eKDIGO criteria which otherwise would be missed by standard KDIGO criteria is quite important and that should be in the main manuscript. The main point of this study is the use of eKDIGO criteria and the added value of deKDIGO criteria for AKI. Hence, I would recommend table showing differences between AKI diagnosed by deKDIGO and KDIGO criteria and deKDIGO and no AKI in main manuscript.*

**This has been added in tables 3 and 4.**

39. *Were there any readmissions? We know AKI patients have high rate of readmissions.  If so, how did authors treat readmission in the analysis? There is risk of survival bias and how did authors take care of this. Interestingly, the mortality reported by authors in much lower than a UK study and I suspect that this is because of inclusion of readmissions (*[*https://doi.org/10.1371/journal.pmed.1003406*](https://doi.org/10.1371/journal.pmed.1003406)*).*

**Please refer to point 23 on the treatment of readmissions and effect of survival bias.**

40. *Can the authors explain why mortality may be lower as the first phase of COVID pandemic was associated with high incidence of AKI and high mortality, especially in UK and Europe.*

**Please refer to point 24 for an explanation on the differences in observed mortality with the cited study.**

41. *I should confess that I am not well versed with GAM and not sure why authors did not perform logistic regression to adjust for the predictors. However, collinearity can still exist in GAM and wonder how did authors check this. Especially, multi-collinearity will exist between mechanical ventilation, cardiac arrest and ICU admission.*

**Please see point 13 for an answer to this.**

42. *Going forward, it will be important to develop artificial intelligence to electronically detect AKI using the extended criteria in electronic patient record*

**We thank the reviewer for this suggestion and agree that it will be an important follow-up study.**

Reviewer #2

"Acute Kidney Injury in Patients with COVID-19 using an Extended KDIGO Definition" promotes the use of an extended Kidney Disease Improving Global Outcomes (KDIGO) definition, to diagnose acute kidney injury (AKI).

The current KDIGO definition depends on whether there is an increase in serum creatinine (sCr) beyond certain thresholds, over 48 hours or within 7 days (Table 1). eKDIGO further includes a decrease in sCr (i.e. deKDIGO) by the same amounts over the same timeframes, as additional criteria. Statistical analysis by pairwise independent samples comparisons adjusting for a number of common confounders suggested that the additionally-identified deKDIGO group would also expect worse outcomes compared to the control group without AKI (sTable5), although not to the extent of the original KDIGO group (sTable4). It is therefore suggested that eKDIGO (which diagnoses the additional deKDIGO group) has the potential to identify more patients with increased
mortality (from AKI), that had not been previously diagnosed by the standard KDIGO definition.

This improved determination of risks appears potentially of great value in addressing the ongoing coronavirus pandemic. However, a number of issues might be considered, most importantly those relating to adjustment for confounders:

43. Given the focus on AKI in the study, it might be considered to further clarify the clinical validity of the proposed additions (i.e. decrease in sCr over time) to the KDIGO criteria. While some prior work ([6],[7]) was cited, it might be considered to explain the reasoning behind a decrease in sCr indicating AKI in slightly more depth in the paper, perhaps with empirical metrics/prior research on (expected) sCr variance in health individuals.

**Please refer to point 25 for a reply to this point.**

44. It is stated that individuals with <2 sCr measurements/tests were excluded, which accounts for n=293,619 of the original n=418,111 individual included in the study. Given that about 70% of the participants has <2 sCr, it might be clarified as to the criteria contributing to the number of sCr taken for each individual. Possible bias of this criteria to the study population might also be discussed further.

**Please refer to point 26 to address this suggestion.**

45. Moreover, the number of sCr measurements/tests taken would also appear to affect the probability that a patient would meet the (e)KDIGO criteria. For example, if a patient has his sCr increase by 2 times over baseline on the third day after admission, but have sCr decrease to just 1.2 times over baseline by the sixth day, then he would be considered to have AKI had he been tested for sCr around the third day (and possibly other days), but not to have AKI had he only been tested for sCr on the sixth day. sCr testing procedure and descriptive statistics relating to the number of sCr tests (i.e. sampling rate), might thus be discussed/presented.

**Please refer to points 26 and 27. S2 Fig in the supplementary material show the number of AKI cases as a proportion of the total number of sCr sampled on each day.**

46. The above clarifications on typical sCr measurement/test schedules would also seem relevant to the presentation of peak AKI stage (Figure 3), since it seems that a patient only has the potential to record a peak AKI on a day when his sCr was taken. Also, it might be clarified as to which day is reflected in Figure 3, if a patient maintaines his peak AKI stage over multiple days (e.g. the first day that the stage is reached)

**Thank you very much for noting this. A clarification of the peak AKI timing in Fig 3 has been added to the Methods section (lines 192 – 194):**

***“For both groups (KDIGO and eKDIGO) time to peak AKI from hospital admission and the respective counts for each day were compared by visual inspection of histograms using the first day that a peak stage was reached”***

47. From the confounders used for adjustment (Table 3), age appears a glaring omission, given how significantly it is known to affect mortality rates in particular (to the extent that it might be fair to say that the results may not be meaningful without it). Is there any reason why it was not considered as a confounder, especially as age is included as part of the demographics (Table 2)? Additionally, stratified analysis by age would seem especially relevant for validation of the findings.

**Age has been added to the logistic regression (Table 5) and analysed in discrete age brackets: 18-65, 65-85 and over 85.**

48. Related to the above, it might be clarified as to how the confounders (Table 3) were chosen, given the number of available and potentially relevant patient features available from the demographics table (Table 2) alone, that were not included as confounders (e.g. obesity, diabetes, etc.)

**Please see point 14 for answer to this.**

49. Since vaccination status would also appear a particularly relevant confounder (for 2021 at least), a brief sensitivity analysis for the period before vaccination became available (if individual vaccination status is unavailable) might be appropriate.

**Please see point 32 for an answer to this**.

50. Brief stratified analyses by country development (i.e. HIC/UMIC/LLMIC) might be considered.

**Please see point 33 for an answer to this**.

51. It is stated that missing values were observed for some variables (as described in sTables 3 to 5). The treatment of missing values in the analysis (e.g. through imputation) might be further clarified.

**A clarification of the treatment of missing values has been added to the methods section (lines 220 - 223):**

***“A logistic regression was fitted to assess the association between eKDIGO AKI with in-hospital mortality and a t-test used to calculate significance. MICE imputation was used to address variable missingness and a sensitivity analysis showing the results without imputation can be found in S4 Table.”***

52. There appears potential to further analyze outcomes according to a flexible (e)KDIGO definition, i.e. describe the change in outcomes as the threshold changes from 0.3ml/dl/48 hours, or >=1.5 times/7 days. This might be considered, perhaps in future work.

**We thank the reviewer for this interesting suggestion. We will certainly consider it in future work.**

Reviewer #3:

In the present manuscript, the authors evaluated the incidence and impact of AKI occurring in COVID-19 patients in the community or early after hospitalization by means of an extended KDIGO definition of AKI which captures a fall in serum creatinine upon admission. They found that among 75670 COVID-19 patients, the incidence of AKI was about two-fold higher when using the extended KDIGO criteria compared to the traditional KDIGO definition. Most of the additional cases identified were stage I AKI. Patients with AKI defined according to extended KDIGO criteria had worse kidney function at admission, more in-hospital complications and increased mortality risk compared to those without AKI.

The following drawbacks are for the Author's consideration:

53. The present study included patients from the ISARIC database with clinically diagnosed or laboratory-confirmed SARS-CoV-2 infection admitted to hospital from February 15th 2020, to February 1st 2021. Actually, the involvement of patients without laboratory-confirmed SARS-CoV-2 infection, but with a clinical diagnosis of COVID-19, which relied on symptoms (e.g., fever, cough, dyspnoea) that could be ascribed to other infectious diseases, may have resulted in the inclusion of participants who were hospitalized for non-COVID-19-related conditions. This issue should be acknowledged as a study limitation. Moreover, Table 2 lists signs and symptoms of patients at hospital admission, but key symptoms that were used to define clinically diagnosed COVID-19 (Table S1), such as cough, dyspnoea, changes in sense of smell and taste, were not provided. These data should be reported in Table 2.

**Please refer to point 34 for an answer to the first part of the question.**

**Symptoms used to clinically diagnose COVID-19 have been added to tables 2, 3 and 4. Loss of taste and loss of smell have not been added given a high degree of missingness in these variables (> 50%) as they were added late to the CRF forms.**

54. According to the information reported in the Statistical analysis section, comparisons between patients with AKI defined according to the extended KDIGO criteria and those without AKI were performed using a conservative Bonferroni adjusted significance level of 5 x 10-5 (Page 8, lines 19-21). However, in Table 2, which provided characteristics of patients with or without AKI defined according to the extended KDIGO criteria, P values lower than 0.001 were outlined. It should be clarified for which of the comparisons provided in Table 2 P value was lower than 5 x 10-5.

**Thank you for noting this. The p-values in tables 2, 3 and 4 have now been changed to reflect the test and significance level used.**

55. The results showed that patients who developed AKI according to the extended KDIGO definition were more likely to require invasive mechanical ventilation and ICU admission compared to those without AKI. In this regard, a recent study found that among patients hospitalized with COVID-19 who required mechanical ventilation and had AKI, 74.5% developed AKI after initiation of mechanical ventilation (Am J Kidney Dis 2021; 77:204-215). Thus, information regarding the temporal relationship of AKI development with initiation of mechanical ventilation and ICU admission should be provided.

**Thank you for this suggestion. Unfortunately, we do not have reliable data on timing of mechanical ventilation and can therefore not report its temporal relationship to AKI.**

56. A logistic Generalized Additive Model was fitted to assess the association between AKI development diagnosed using extended KDIGO criteria with in-hospital mortality, adjusting for gender, requiring ICU admission and/or invasive mechanical ventilation, complications and treatment with corticosteroids or antifungal agents. However, several covariates which may have affected the risk of in-hospital mortality were not included in the model, such as ethnicity, with Black race having been reported to strengthen the association between AKI and the risk of death among patients hospitalized with COVID-19 (Clin J Am Soc Nephrol 2020; 16:14-25), and co-morbidities (e.g., chronic kidney disease). Moreover, the relationship between AKI diagnosed by extended KDIGO criteria and in-hospital mortality should also be described using a Kaplan-Meier survival curve.

**We thank the reviewer for these valuable suggestions. Please refer to point 31 and 35 as well as the updated Table 5 (logistic regression) for a reply to the first two aspects.**

**With regards to the final comment, we agree that the relationship between eKDIGO AKI and in hospital mortality should be further analysed. However, we propose using the Aalen – Johansen estimator in place of the Kaplan-Meier estimator to approximate the survival functions. We consider a 28 day follow up window, beginning on the day of hospital admission and ending on either the day of discharge or death, or 28 days post admission if there had not yet been an event occurrence.**

**Use of the KM estimator would require the following conditional probability statement to hold:**

**P(death | discharge) = P(death | still admitted).**

**It’s clear in our population that this doesn’t hold. By using the AJ estimator we can separately model our two competing outcomes to obtain a calibrated estimate of the survival function.**

**We have added this as Figure 4 in the main manuscript.**

57. In the Discussion it was argued that the considerable proportion of the study population from low- and middle-income countries afforded great generalizability of the study findings (Page 16, lines 16-18). Nevertheless, only about 5.3% of the overall patient population was from low- and low middle-income countries (i.e., 4037 out of 75670). Moreover, although 54 countries were involved in the present study, based on data in Figure S1 it can be inferred that more than half of the countries contributed with less than 35 patients with AKI defined according to serum creatinine criteria. Thus, the conclusions regarding the generalizability of the study findings should be tempered.

**Thank you. The statement on country income generalizability has been removed.**

58. It would be valuable to assess the temporal variation of AKI rate, defined according to the traditional and the extended KDIGO definitions, during the observation period, in light of previous studies which suggested a fall in AKI incidence among COVID-19 patients following the first pandemic wave (Nephrol Dial Transplant 2021; doi: 10.1093/ndt/gfab303; Clin J Am Soc Nephrol 2020; 16:14-25; Kidney Int Rep 2021; 6:916-927).

**While this is an interesting suggestion, this analysis was beyond the scope of our work.**

59. The proportion of patients who achieved recovery of kidney function by the time of hospital discharge among surviving patients who had developed AKI, defined according to the traditional and the extended KDIGO definitions, should be provided.

**Please refer to point 37 for an answer to this comment.**

Minor points:

60. According to the flowchart of the study (Figure 1) individuals with laboratory confirmed SARS-CoV-2 infection admitted to hospital from February 15th 2020 to February 1st 2021 were included in the present study. It should be specified that also patients with clinically diagnosed COVID-19 were included.

**This has been corrected. Thank you.**

61. Several inconsistencies throughout the manuscript should be corrected. In particular:
    a. Sixty countries were involved in the study based on the information in the Abstract, but they were 54 according to data in the Results section and in Figure 1;

**It should be 54 countries. This has been corrected in the abstract.**

b. 12740 patients were identified as having AKI according to the traditional KDIGO definition based on the information in the Results section, but they were 12704 according to data in Figure 2 and in Table S4;

**Thank you. This has been corrected in the Results section (the correct number is 12704 pts)**

c. 23982 patients were identified as having AKI according to the extended KDIGO definition based on the information in the Results section, but they were 23892 according to data in Figure 2 and in Table 2;

**This has been corrected.**

d. Patients with AKI who required acute dialysis were 4262 according to the information in the Results section, whilst they were 4252 based on data in Table

**This has been corrected.**

e. the estimated incidence of AKI according to the traditional KDIGO definition was 18% according to the information in the Discussion (Page 14, line 10), but it was 16.8% throughout the manuscript.

**This has been corrected in the discussion.**

62. Regarding laboratory parameters, it is unclear whether urea levels were reported as either blood urea concentrations or blood urea nitrogen, and if median values were expressed as mg/dL (as indicated in Table 2) or as mmol/L (as indicated in the main text). As for kidney function, the equation used to estimate glomerular filtration rate (eGFR) should be specified, and it should be clarified whether the reported eGFR values were indexed for body surface area (as reported in the core paper) or not indexed for body surface area (as reported in Table 2).

**Thank you for noting this. The inconsistencies have been corrected.**

63. The last sentence of the paragraph entitled "Demographic and clinical characteristics" is incomplete.

**This has been corrected, thank you.**

64. Figure S1 was never cited throughout the manuscript; this should be done.

**This has been added to the results section.**

**References**

1.	Sullivan MK, Lees JS, Drake TM, Docherty AB, Oates G, Hardwick HE, et al. Acute kidney injury in patients hospitalized with COVID-19 from the ISARIC WHO CCP-UK Study: a prospective, multicentre cohort study. Nephrology Dialysis Transplantation. 2022;37(2):271-84.

2.	Kolhe NV, Fluck RJ, Selby NM, Taal MW. Acute kidney injury associated with COVID-19: A retrospective cohort study. PLoS medicine. 2020;17(10):e1003406.

3.	Bowe B, Cai M, Xie Y, Gibson AK, Maddukuri G, Al-Aly Z. Acute kidney injury in a national cohort of hospitalized US veterans with COVID-19. Clinical Journal of the American Society of Nephrology. 2021;16(1):14-25.

4.	Chan L, Chaudhary K, Saha A, Chauhan K, Vaid A, Zhao S, et al. AKI in hospitalized patients with COVID-19. Journal of the American Society of Nephrology. 2021;32(1):151-60.

5.	Hirsch JS, Ng JH, Ross DW, Sharma P, Shah HH, Barnett RL, et al. Acute kidney injury in patients hospitalized with COVID-19. Kidney International. 2020;98(1):209-18.

6.	Ng JH, Hirsch JS, Hazzan A, Wanchoo R, Shah HH, Malieckal DA, et al. Outcomes among patients hospitalized with COVID-19 and acute kidney injury. American Journal of Kidney Diseases. 2021;77(2):204-15. e1.

7.	Kidney Disease: Improving Global Outcomes (KDIGO) Acute Kidney Injury Workgroup. KDIGO Clinical practice guideline for acute kidney injury. Kidney Int Suppl. 2012;2(1):1-138.

8.	Mehta RL, Burdmann EA, Cerdá J, Feehally J, Finkelstein F, García-García G, et al. Recognition and management of acute kidney injury in the International Society of Nephrology 0by25 Global Snapshot: a multinational cross-sectional study. The Lancet. 2016;387(10032):2017-25.

9.	Yang L, Xing G, Wang L, Wu Y, Li S, Xu G, et al. Acute kidney injury in China: a cross-sectional survey. The Lancet. 2015;386(10002):1465-71.

**References**

1.	Hirsch JS, Ng JH, Ross DW, Sharma P, Shah HH, Barnett RL, et al. Acute kidney injury in patients hospitalized with COVID-19. Kidney International. 2020;98(1):209-18.

2.	Ng JH, Hirsch JS, Hazzan A, Wanchoo R, Shah HH, Malieckal DA, et al. Outcomes among patients hospitalized with COVID-19 and acute kidney injury. American Journal of Kidney Diseases. 2021;77(2):204-15. e1.

3.	Sullivan MK, Lees JS, Drake TM, Docherty AB, Oates G, Hardwick HE, et al. Acute kidney injury in patients hospitalized with COVID-19 from the ISARIC WHO CCP-UK Study: a prospective, multicentre cohort study. Nephrology Dialysis Transplantation. 2022;37(2):271-84.

4.	Bowe B, Cai M, Xie Y, Gibson AK, Maddukuri G, Al-Aly Z. Acute kidney injury in a national cohort of hospitalized US veterans with COVID-19. Clinical Journal of the American Society of Nephrology. 2021;16(1):14-25.

5.	Chan L, Chaudhary K, Saha A, Chauhan K, Vaid A, Zhao S, et al. AKI in hospitalized patients with COVID-19. Journal of the American Society of Nephrology. 2021;32(1):151-60.

6.	Mehta RL, Burdmann EA, Cerdá J, Feehally J, Finkelstein F, García-García G, et al. Recognition and management of acute kidney injury in the International Society of Nephrology 0by25 Global Snapshot: a multinational cross-sectional study. The Lancet. 2016;387(10032):2017-25.

7.	Ostermann M, Bellomo R, Burdmann EA, Doi K, Endre ZH, Goldstein SL, et al. Controversies in acute kidney injury: conclusions from a Kidney Disease: Improving Global Outcomes (KDIGO) Conference. Kidney International. 2020;98(2):294-309. doi: 10.1016/j.kint.2020.04.020.

8.	Selby NM, Fluck RJ, Kolhe NV, Taal MW. International Criteria for Acute Kidney Injury: Advantages and Remaining Challenges. PLOS Medicine. 2016;13(9):e1002122. doi: 10.1371/journal.pmed.1002122.

9.	Siew ED, Matheny ME, Ikizler TA, Lewis JB, Miller RA, Waitman LR, et al. Commonly used surrogates for baseline renal function affect the classification and prognosis of acute kidney injury. Kidney international. 2010;77(6):536-42.

10.	Kidney Disease: Improving Global Outcomes (KDIGO) Acute Kidney Injury Workgroup. KDIGO Clinical practice guideline for acute kidney injury. Kidney Int Suppl. 2012;2(1):1-138.
